# The relation between household income and surgical outcome in the Dutch setting of equal access to and provision of healthcare

**DOI:** 10.1371/journal.pone.0191464

**Published:** 2018-01-22

**Authors:** Klaas H. J. Ultee, Elke K. M. Tjeertes, Frederico Bastos Gonçalves, Ellen V. Rouwet, Anton G. M. Hoofwijk, Robert Jan Stolker, Hence J. M. Verhagen, Sanne E. Hoeks

**Affiliations:** 1 Department of Surgery, Erasmus University Medical Center, Rotterdam, the Netherlands; 2 Department of Anaesthesiology, Erasmus University Medical Center, Rotterdam, the Netherlands; 3 Department of Surgery, Hospital de Santa Marta, Centro Hospitalar de Lisboa Central, Lisbon, Portugal; 4 Department of Surgery, Zuyderland, Sittard, the Netherlands; University of Kentucky, UNITED STATES

## Abstract

**Background:**

The impact of socioeconomic disparities on surgical outcome in the absence of healthcare inequality remains unclear. Therefore, we set out to determine the association between socioeconomic status (SES), reflected by household income, and overall survival after surgery in the Dutch setting of equal access and provision of care. Additionally, we aim to assess whether SES is associated with cause-specific survival and major 30-day complications.

**Methods:**

Patients undergoing surgery between March 2005 and December 2006 in a general teaching hospital in the Netherlands were prospectively included. Adjusted logistic and cox regression analyses were used to assess the independent association of SES–quantified by gross household income–with major 30-day complications and long-term postoperative survival.

**Results:**

A total of 3929 patients were included, with a median follow-up of 6.3 years. Low household income was associated with worse survival in continuous analysis (HR: 1.05 per 10.000 euro decrease in income, 95% CI: 1.01–1.10) and in income quartile analysis (HR: 1.58, 95% CI: 1.08–2.31, first [i.e. lowest] quartile relative to the fourth quartile). Similarly, low income patients were at higher risk of cardiovascular death (HR: 1.26 per 10.000 decrease in income, 95% CI: 1.07–1.48, first income quartile: HR: 3.10, 95% CI: 1.04–9.22). Household income was not independently associated with cancer-related mortality and major 30-day complications.

**Conclusions:**

Low SES, quantified by gross household income, is associated with increased overall and cardiovascular mortality risks among surgical patients. Considering the equality of care provided by this study setting, the associated survival hazards can be attributed to patient and provider factors, rather than disparities in healthcare. Increased physician awareness of SES as a risk factor in preoperative decision-making and focus on improving established SES-related risk factors may improve surgical outcome of low SES patients.

## Introduction

The relation between socioeconomic status (SES) and outcome of medical treatment has been the subject of many studies over the past years, and SES-related risks of poor outcome have been demonstrated previously.[1–9] A considerable number of these studies were performed in countries where healthcare is not publicly provided. Although the relation between SES and outcome is multifactorial and complex, differences in outcome between socioeconomic classes were attributed more to differences in accessibility and provision of care in some of these studies, rather than patient factors or healthcare provider factors.[1, 6, 9–11]

As a result of governmental regulation, medical care in the Netherlands is equal among all layers of society, and has even been credited the most equally accessible healthcare system in the world.[12, 13] This characteristic of the present study setting provides a new and unique opportunity to assess the role of SES on outcome of care. Due to the healthcare equality, differences in outcome associated with SES can under these circumstances be attributed to patient and provider factors and their interaction, rather than disparities in healthcare. We have previously demonstrated in a vascular surgery population that SES–quantified by gross household income–implicated significant postoperative survival risks, independent from conventional medical and environmental risk factors.[14] These findings suggest that SES encompasses a wide variety of risk factors and behaviors that are not adequately captured by conventionally considered risk factors.

The association between SES and prognosis in a non-vascular general surgical population remains unexplored. Moreover, it is well known that vascular disease and vascular patients are relatively more susceptive to environmental risk factors, which limits the generalizability of the previous study to non-vascular patients.

The primary objective of this study is to determine the association between SES, reflected by household income, and survival after surgery in a general surgical population. Additionally, we aim to establish whether SES is associated with cause-specific survival and major 30-day complication

## Patients and methods

### Study population

Patients undergoing elective or acute surgery between March 2005 and December 2006 in a medium-sized general teaching hospital in the Netherlands were prospectively included.[15] Procedures are detailed in S1 Table. Since the association between low household income and worse outcome among vascular surgery patients has been established in the previous study,[14] vascular procedures were excluded. Additional exclusion criteria were surgical interventions performed under local anesthesia, and patients younger than 14 years at the time of the procedure. Bariatric surgery was not performed in this hospital. When a patient underwent multiple surgical procedures within the study period, the first operation was included for analysis and survival was assessed from that moment onward. The institutional review board of Zuyderland Medical Center approved this study, and patient consent was waived due to the de-identified nature of the data. The study complies with the Helsinki declaration on research ethics.

### Baseline characteristics

Medical characteristics were obtained by a surgeon or a surgical resident during a routine visit prior to surgery. Pulmonary disease was defined as an illness of the lung or respiratory system (i.e. asthma, lung cancer, chronic infections, previous pulmonary embolisms, chronic obstructive pulmonary disease (COPD)). Cardiac disease was considered when the medical history included coronary artery disease (with or without coronary revascularization), heart failure, arrhythmias, valvular heart disease or cardiomyopathy. Cerebrovascular disease was defined as either a Transient Ischemic Attack (TIA) or ischemic stroke in the medical history. A patient was considered diabetic when diabetes mellitus was mentioned in the prior history or medical records show use of insulin or oral anti-diabetics. Hypertension was considered when hypertensive disease was mentioned in the medical history or the patient received anti-hypertensive medication. A history of cancer was defined as malignant neoplastic disease in the prior medical history.

Gathered surgery-related data included the type of anesthesia (locoregional or general) and the surgical setting (inpatient or outpatient). The risk of the performed procedure was defined as low, intermediate or high risk conform the surgical risk classification system by Boersma et al. (S1 Table).[16] High-risk surgical procedures solely consist of major vascular procedures and were not included in this study for previously mentioned reasons. Finally, all events following surgery were documented. A surgical resident as well as a member of the surgical staff independently scored all complications. To ensure complications were interpreted objectively and systematically, a classification proposed by Clavien et al. was used as guidance.[17] A major complication was defined as a complication requiring surgical, endoscopic or radiological intervention with or without residual organ dysfunction. Validation of the database using a random sampling audit procedure confirmed a high level of accuracy and completeness of the data.

### Endpoints

The primary endpoint was overall mortality. Secondary endpoints were major 30-day complications, cardiovascular and cancer-related mortality.

### Socioeconomic status

In this study, gross household income was used as an indicator of SES. Household income is one of the most widely accepted and used methods to quantify SES, and has previously been affirmed to provide an accurate reflection of SES-related health disparities.[18–20] To avoid missing income data due to a patients’ death in the year of surgery, gross household income in the year prior to the year of surgery was used to quantify SES. Annual earnings were obtained at the Dutch Central Bureau of Statistics (CBS), and encompassed all types of income of people sharing a household or place of residence combined, including salary, (state) pension, social compensation, and investment revenues. Patients were assigned income percentiles and quartiles in accordance with the national income distribution. To clarify, first income quartile patients included members of a household with an annual salary that corresponds to 0–25% gross household incomes of the Dutch population.

### Cause of death

Causes of death obtained through national death registries, which are also maintained by the CBS. The high accuracy of Dutch cause-of-death registration has been demonstrated previously.[21] The cause of death was defined as the cause for the initial health deterioration, which subsequently resulted in death. This approach is similar to the strategy employed for the overall Dutch population death registrations and reports. Autopsy was not routinely performed. The causes of death were coded in accordance with *International Classification of Diseases*, *10th Revision* (ICD-10). Cardiovascular death was defined as I10-I79, and cancer-related death as C00-C43, C45-C97.

To obtain information on household income and causes of death, a database consisting of medical data on all study participants was anonymised and matched to the household income and death registry data sets maintained by the CBS. Dutch privacy legislation stipulates that data analysis with national data is only allowed by authorized researchers (KU, FBG) from designated institutions inside a secure environment after approval from the institutional ethical committee. Furthermore, output was checked by the CBS for privacy violations before it was allowed for publication purposes.

### Statistical methods

Baseline characteristics are presented as counts and percentages (dichotomous variables), means and standard deviations (continuous variables), or medians and interquartile ranges (IQR). Patients were grouped in quartiles in correspondence with the national gross household income distribution. Differences at baseline between income quartiles were tested using Pearson’s chi-square analysis and ANOVA, where appropriate. The predictive value of household income for long-term survival was assessed using Cox-regression analysis. In order to determine both the type (i.e. linear or exponential) and the clinical significance of the relation between income and survival, analyses were performed with income as a continuous variable as well as categorical per income quartile. Exponential properties were tested by including higher-order terms of income in the regression model in continuous analysis. In income quartile analysis, the highest income quartile was designated reference category. Multivariable analyses were performed in a stepwise manner. The step 1 multivariable model adjusted for: surgical risk, age, gender, diabetes, hypertension, cerebrovascular disease, cardiac disease, malignant disease, pulmonary disease. The step 2 multivariable model additionally adjusted for: smoking and BMI. Cause specific mortality hazards (i.e. cardiovascular and cancer-related) associated with household income were established with the same Cox model. The association between income and major 30-day complications and death following surgery was studied using logistic regression analysis. The multivariable model consisted the same covariates as the long-term survival models. Sensitivity analyses were performed to assess whether the association between income and postoperative survival existed among all patients, including vascular patients. All tests were two-sided and significance was considered when P-value <0.05. Statistical analysis was performed using the IBM SPSS Statistics 20 (IBM Inc., Chicago, IL).

## Results

A total of 4153 patients were suitable for analysis. The gross household income could be retrieved for 3929 patients (94.6%).

### Baseline characteristics

Baseline characteristics are detailed in Table 1. Low household income patients were younger (P<0.001) and were more frequently female (P<0.001). All medical conditions were more common among lower income quartile patients (P<0.001 for all medical conditions). Similarly, higher income patients were less often current or former smokers (P<0.001). BMI also significantly differed between the income quartiles (P<0.001).

**Table 1:** Baseline characteristics.

	Quartile 1 (n = 708)	Quartile 2 (n = 1122)	Quartile 3 (n = 1083)	Quartile 4 (n = 1016)	P-value
**Demographics**					
*Age—mean (± SD)*	61.8 (19.4)	59.3 (16.5)	48.6 (15.6)	46.9 (14.5)	<0.001
*Female gender—n (%)*	435 (61)	538 (48)	525 (48)	446 (44)	<0.001
**Comorbid conditions**					
*Diabetes mellitus—n (%)*	91 (13)	96 (9)	68 (6)	45 (4)	<0.001
*Hypertension—n (%)*	189 (27)	242 (22)	160 (15)	119 (12)	<0.001
*Cerebrovascular disease—n (%)*	67 (10)	87 (8)	39 (4)	10 (<1)	<0.001
*Cardiac disease—n (%)*	184 (26)	239 (21)	131 (12)	76 (8)	<0.001
*Malignant disease—n (%)*	218 (31)	321 (29)	223 (21)	184 (18)	<0.001
*Pulmonary disease—n (%)*	128 (18)	197 (18)	124 (12)	79 (8)	<0.001
**Surgical risk**					
*Low—n (%)*	363 (51)	653 (58)	681 (63)	671 (66)	<0.001
*Intermediate—n (%)*	345 (49)	469 (42)	402 (37)	345 (34)	<0.001
**Behavioral risk factors**					
*Smoking* *—*n (%)*	236 (46)	431 (51)	428 (52)	284 (39)	<0.001
*BMI—mean (± SD)*	26.1 (4.7)	26.2 (4.4)	26.5 (4.8)	25.7 (4.3)	0.004
**Type of anesthesia**					
*General—n (%)*	618 (87)	936 (84)	920 (85)	855 (84)	0.135
**Socioeconomic status**					
*Median income*—*€ (IQR)*	16 620.50 (13 914.25–19 280.75)	29 375.50 (25 119.50–34 474.75)	50 971.00 (44 961.00–57 645.00)	83 490.50 (72 924.50–101 192.75)	-

* approximately 25% missing values; IQR: interquartile range

### Major 30-day complications

In the first 30 days following surgery, 206 patients suffered a major complication requiring additional interventions (either surgical, endoscopic or radiological) (Table 2). Within this group, 37 patients (18%) were left with residual organ dysfunction. Income was associated with the occurrence of major complications in univariate continuous analysis (OR: 1.05, 95% CI: 1.004–1.11), as well as in income quartile analysis for the first quartile (OR: 1.99, 95% CI: 1.30–3.04) compared to the fourth quartile (Table 3). However, no association could be established in adjusted analysis.

**Table 2:** Survival and short- and long-term event characteristics in accordance with household income quartiles.

	Quartile 1 (n = 708)	Quartile 2 (n = 1122)	Quartile 3 (n = 1083)	Quartile 4 (n = 1016)	Total (n = 3929)	P-value
*5-year survival estimate (± se)*	77% (1.6)	84% (1.1)	91% (0.9)	96% (0.6)	88% (0.5)	<0.001
*Median follow-up—years* *(IQR)*	6.2 (5.2–6.7)	6.3 (5.8–6.7)	6.4 (5.9–6.8)	6.4 (5.9–6.8)	6.3 (5.8–6.8)	-
**Endpoints**						
*Severe complications—n (%)*	52 (7)	61 (5)	54 (5)	39 (4)	206 (5)	0.014
*Overall death—n (%)*	189 (27)	222 (20)	107 (10)	52 (5)	570 (15)	<0.001
*Cardiovascular death—n (%)*	54 (8)	38 (3)	11 (1)	5 (<1)	108 (3)	<0.001
*Cancer-related death—n (%)*	71 (10)	117 (10)	60 (6)	33 (3)	281 (7)	<0.001

**Table 3:** The association between household income and major 30-day complications following surgery.

	Continuous	Quartile 1	Quartile 2	Quartile 3	Quartile 4
Major complications					
*Univariate*	1.05 (1.004–1.11)	1.99 (1.30–3.04)	1.44 (0.96–2.17)	1.32 (0.86–2.00)	-
*Multivariate step 1*	0.99 (0.95–1.03)	1.07 (0.66–1.73)	0.89 (0.57–1.39)	1.18 (0.76–1.81)	-
*Multivariate step 2*	1.01 (0.95–1.06)	1.09 (0.62–1.92)	1.02 (0.61–1.70)	1.41 (0.86–2.31)	-

Odds ratios in continuous analyses are determined per 10.000 euro decrease in household income. In quartile analyses, the fourth quartile serves as reference category. Step 1 multivariable analysis adjusted for: surgical risk, age, gender, diabetes, hypertension, cerebrovascular disease, cardiac disease, malignant disease and pulmonary disease. Step 2 multivariable analysis additionally adjusted for: smoking and BMI.

### Overall mortality

During a median follow-up of 6.3 years 570 deaths occurred (Table 2). Regarding the relation between income and overall survival, a significant association was found in continuous analysis (Table 4). In multivariable step 1, as well as adjusted for behavioral risk factors in step 2, mortality hazards proved to increase as income diminished (HR: 1.05 per 10.000 euro decrease in household income, 95% CI: 1.01–1.10,). A similar relation was found in income quartile analysis. In step 2 multivariable analysis, patients in the first quartile (i.e. the lowest income quartile) had significantly higher mortality risks (HR: 1.58, 95% CI: 1.08–2.31). The association lost significance in the second and third quartile, although a trend remained (HR: 1.41, 95% CI: 0.99–2.02, HR: 1.32, 95% CI: 0.90–1.93, respectively for the second and third quartile).

**Table 4:** The association between household income and overall mortality.

	Continuous	Quartile 1	Quartile 2	Quartile 3	Quartile 4
Overall mortality					
*Univariate*	1.25 (1.21–1.30)	5.89 (4.33–8.00)	4.17 (3.08–5.64)	1.97 (1.41–2.74)	-
*Multivariate step 1*	1.06 (1.01–1.10)	1.49 (1.06–2.09)	1.40 (1.02–1.93)	1.30 (0.93–1.83)	-
*Multivariate step 2*	1.05 (1.01–1.10)	1.58 (1.08–2.31)	1.41 (0.99–2.02)	1.32 (0.90–1.93)	-

Hazard ratios in continuous analyses are determined per 10.000 euro decrease in household income. In categorical analyses, the fourth quartile serves as reference category. Step 1 multivariable analysis adjusted for: surgical risk, age, gender, diabetes, hypertension, cerebrovascular disease, cardiac disease, malignant disease and pulmonary disease. Step 2 multivariable analysis additionally adjusted for: smoking and BMI.

### Cause specific mortality

Of the 570 deaths, 108 (19%) were due to cardiovascular causes. In both step 1 and step 2 continuous analysis, low household income was significantly associated with increased cardiovascular mortality risks (HR: 1.26 per 10.000 euro decrease in household income, 95% CI: 1.07–1.48, Table 5). In income quartile analysis, a significant independent income-related cardiovascular survival hazard was observed in the first quartile (HR: 3.10, 95% CI: 1.04–9.22). No relation could be established for the higher two quartiles.

**Table 5:** The association between household income and cause-specific mortality.

	Continuous	Quartile 1	Quartile 2	Quartile 3	Quartile 4
**Cardiovascular**					
*Univariate*	1.41 (1.33–1.51)	17.99 (7.20–44.97)	7.59 (2.99–19.29)	2.11 (0.73–6.08)	-
*Multivariate step 1*	1.22 (1.09–1.37)	2.84 (1.08–7.50)	1.79 (0.69–4.65)	1.19 (0.41–3.46)	-
*Multivariate step 2*	1.26 (1.07–1.48)	3.10 (1.04–9.22)	1.40 (0.47–4.20)	1.17 (0.36–3.86)	-
**Cancer-related**					
*Univariate*	1.19 (1.13–1.24)	3.46 (2.29–5.23)	3.43 (2.33–5.05)	1.74 (1.14–2.66)	-
*Multivariate step 1*	1.04 (0.99–1.10)	1.28 (0.81–2.02)	1.42 (0.95–2.14)	1.30 (0.85–2.01)	-
*Multivariate step 2*	1.01 (0.96–1.06)	1.04 (0.63–1.72)	1.40 (0.90–2.18)	1.36 (0.86–2.15)	-

Hazard ratios in continuous analyses are determined per 10.000 euro decrease in household income. In categorical analyses, the fourth quartile serves as reference category. Step 1 multivariable analysis adjusted for: surgical risk, age, gender, diabetes, hypertension, cerebrovascular disease, cardiac disease, malignant disease and pulmonary disease. Step 2 multivariable analysis additionally adjusted for: smoking and BMI.

Cancer-related death was ascertained in 281 (49%) cases. In continuous analysis, a significant relation was found between income and cancer-related survival in univariate analysis (HR: 1.19, 95% CI: 1.13–1.24). The relation was lost after adjusting for conventional risk estimators in multivariable analysis. Similarly, lower quartile patients were not burdened by additional cancer-related mortality in multivariable income quartile analysis.

### Sensitivity analyses

Sensitivity analyses with vascular surgery patients included showed that household income was associated with worse overall survival in continuous step 2 multivariable analysis (HR: 1.05 per 10.000 euro decrease in household income, 95% CI: 1.01–1.09,), as well as cardiovascular survival (HR: 1.21, 95% CI: 1.02–1.41), while no increased risk was found for cancer-related survival (HR: 1.01, 95% CI: 0.96–1.07). Income quartile analyses showed similar results for overall and cancer-related mortality as well. For cardiovascular mortality, a non-significant trend towards increased cardiovascular survival hazards was observed among first quartile patients (P = 0.055).

## Discussion

The principal finding of this study is that SES, reflected by household income, is a significant predictor of long-term survival in an overall surgical population. Cause specific mortality analysis indicated that the mortality hazards associated with low household income were not caused by increased risks of death due to cancer-related causes, but rather a higher risk of cardiovascular death. Since the association maintained after adjusting for demographics, comorbidities and behavioral risk factors, the mortality risks add to conventionally considered risk estimators. Secondly, this study showed that SES is not related to short-term postoperative outcome, as demonstrated by the lack of association with major 30-day complications.

Differences in outcome after surgery between socioeconomic classes have previously been attributed to disparities in quality and provision of care.[1, 6, 9, 22, 23] However, the equality in access to and provision of care provided by this study setting suggests that not healthcare inequalities, but rather patient-related factors that are not adequately captured by conventionally considered risk factors played a dominant causal role in SES-related outcome differences. Hence, even in countries where healthcare is not publicly provided, differences in healthcare utilization are unlikely to fully account for divergences in outcome.[24, 25] This is in line with a report by Kilbourne et al., which introduced a model on the determinants of healthcare disparities.[11] Kilbourne et al. propose that healthcare disparities originate from individual, provider, and healthcare system factors. While the impact of disparities in healthcare system factors may be minimal in The Netherlands, individual and provider factors, and their interaction, are likely to be of influence.

With regard to individual patient factors, it has been reported that less than 50% of socioeconomic differences in disease occurrence and prognosis are explained by combined common behavioral risk factors, such as smoking.[19, 26–28] What patient-related factors may drive the association of low SES with worse outcome? First, socioeconomic disadvantage is a known risk factor for poor compliance to medication, diet, and lifestyle restrictions.[29–33] Second, psychosocial risk factors implicated in the etiology of cardiovascular disease, such as psychological stress, depression and social isolation, are more often observed in low SES populations.[34–37] Also, material deprivation in individuals from disadvantaged backgrounds is associated with worse dietary quality.[38–41] In addition, SES has been has been established as an important determinant of physical activity and exercise,[42] which–in turn–is associated with health status and life-expectancy.[42, 43] Fifth, low SES patients tend to reside in more disadvantaged neighborhoods with higher concentrations of harmful air pollutants and worse housing conditions, which are associated with worse health outcomes.[44–46] Physical demand, low decision latitude and high job strain, which are more common in lower employment grades, may also explain some of the excess risk among disadvantaged groups.[47]

These factors have been linked to especially increased risks of cardiovascular disease and mortality.[28, 46, 48–50] Moreover, literature based models suggest that perhaps even epigenetical factors among lower socioeconomic classes may be responsible for the higher prevalence of cardiovascular disease among lower socioeconomic classes.[51] This provides a valid explanation as to why low SES predominantly implied cardiovascular survival hazards in our study.[52, 53] Although no relation between SES and cancer-related death was found in the full model, studies have proven such relation to exist.[54, 55] Our results showed an association between SES and cancer-related mortality in univariate analysis, but no relation could be established when adjusting for conventional risk factors.[55] This is in line with previous studies showing that that much of the SES-related risk of cancer occurrence and mortality are through conventional risk factors, most importantly smoking.[52, 56–58]

Apart from patient-related factors, the previously mentioned provider factors, and their interaction with patient factors, may also influence the relation between SES and poor outcome.[11] Particularly stereotyping of patients with different cultural or ethnic backgrounds and problems in communication between patient and provider play an important role.[59–63] Aside from causing suboptimal care,[60] the discrepancies may result in mistrust and lack of patient engagement in treatment, which only further promote SES-related health disparities.[61, 62, 64] Although the association between low SES and worse outcome is multifactorial and complex, a better understanding of the relation between low SES and worse outcome may help to attenuate health disparities. In addition to focus on bettering SES-related patient factors, increased physician awareness and improvement of communication between patient and provider may help to improve outcome of low SES surgical patients.[64]

In regards to the association between SES and major complications following surgery, a relation was found in univariate analysis, but point estimates decreased to 1 and significance was lost in the multivariable model. The fact that the relation did not maintain significance after adjusting for commonly considered health hazards suggests that SES is merely a proxy measure in this association and that it provides no additional value over conventional risk factors for the prediction of the short-term postoperative course.

This study has some limitations that should be considered. First of all, it should be noted that only patients who underwent surgery were included. Patients who were conservatively treated and those with prohibitive surgical risks due to severe comorbidity were consequently excluded. In addition, smoking status was unobtainable for a considerable amount of patients, and resulted in the exclusion of approximately 25% of cases in the full model. Although healthcare in the Netherlands has been established as equal among different layers of society, it would have been valuable to assess the association between socioeconomic status and the various parameters of access to and quality of healthcare. Unfortunately, our data provides insufficient detail to comment on the impact of household income on the different aspects of access and quality of care, and potential interactions. Finally, American studies that have reported on SES-related outcome and healthcare disparities often describe divergences between racial groups as well. Due to Dutch legislation, documentation of ethnicity in patient records is only allowed when medically relevant. Consequently, racial disparities could unfortunately not be investigated.

In conclusion, this study demonstrates that low household income, as an indicator of low SES, is a risk factor for overall and cardiovascular mortality following surgery. Considering the equality in access to and provision of healthcare provided by this study setting, the present results suggest that the observed health hazards accompanying low socioeconomic status are likely to be caused by patient and provider factors, rather than differences in medical care. Although the exact mechanism mediating the postoperative SES-related survival risk remains unclear, increased physician awareness and improvement of known SES-related risk factors and behaviors may help to improve surgical outcome among low SES patients.

## Supporting information

S1 Table Risk classification of included surgical procedures. (DOC)
