# Supplementary material for: The relation between household income and surgical outcome in the Dutch setting of equal access to and provision of healthcare
Source: PLoS One. 2018 Jan 22;13(1):e0191464. doi: 10.1371/journal.pone.0191464 (PMC5777644; doi:10.1371/journal.pone.0191464)
Supplement: S1 Table — (DOC) [file pone.0191464.s001.doc]

**Supplemental Table 1.** Risk classification of included surgical procedures

| **Low risk surgery** | **Procedure** |
| --- | --- |
|  | Hernia surgery (except incisional hernia surgery) |
|  | Varicose vein surgery |
|  | Perianal surgery |
|  | Minor trauma surgery |
|  | Minor surgery of soft tissue |
| **Intermediate risk surgery** | **Procedure** |
|  | Appendectomy |
|  | Cholecystectomy |
|  | Major abdominal surgery (i.e. liver, gastric, bowel, spleen esophagus, incisional hernia surgery) |
|  | Head and neck surgery |
|  | Thoracic surgery |
|  | Major trauma surgery (i.e. multitrauma or trauma involving the femur or hip) |
